# Determination of exposure to lead of subjects from southwestern Poland by human hair analysis

**DOI:** 10.1007/s10661-013-3534-3

**Published:** 2013-12-18

**Authors:** Izabela Michalak, Paulina Wołowiec, Katarzyna Chojnacka

**Affiliations:** Institute of Inorganic Technology and Mineral Fertilizers, Wroclaw University of Technology, Smoluchowskiego 25, 50-372 Wroclaw, Poland

**Keywords:** Lead, Mineral analysis of scalp hair, Environmental exposure, Lifestyle, Dietary habits

## Abstract

The aim of the present work was to investigate the exposure to lead from various sources by investigation of mineral composition of human scalp hair. The research was carried out on hair sampled from 267 young adults living in Wrocław (southwest Poland). The effect of the place of residence, diet, and lifestyle on lead content in hair was examined by inductively coupled plasma optical emission spectrometry (ICP-OES). Lead was determined at the wavelength 220.353 nm. These outcomes were reached by linking the results of lead level in hair with the results of questionnaire survey. The mean lead level in hair of the whole examined population was 2.01 ± 2.10 mg kg^−1^. Lead can enter the human body mainly by inhalation and gastrointestinal absorption. It was found that consuming cheese, fish, and lettuce caused increased level of lead in hair. On the other hand, drinking of milk, tea, coffee, or lemon resulted in decreased content of lead in hair. Additional source of exposure to lead could be cigarette smoking, distance to the traffic road, painting the walls, amalgam filling. Based on the results, it can be concluded that exposure to lead can occur mainly from eating habits and environmental exposure.

## Introduction

Despite successful programs undertaken to lower the level of lead cycling in the environment, human exposure to this toxic metal remains of concern to public health (WHO 1995; Li et al. 2013). Lead is ubiquitous in human environment and is widely used in many industrial processes (D’souza et al. 2011). The major sources of lead pollution are mining and smelting of lead ores, refining and processing of compounds, lead-lined food and drink containers, waste incineration, industrial plants using coal, lead-contaminated soil, and lead-based paint (Ziemacki et al. 1989; Mielkel and Reagan 1998; Flora et al. 2013). Exposure of the general population to lead occurs mainly through the ingestion of contaminated food and drinking water and by the inhalation of particulate lead present in air (D’souza et al. 2011). In the work of Pizzol et al. (2010), it was confirmed that the critical route of exposure to lead was via ingestion, accounting for 99 % of total lead intake, while inhalation contributed only to 1 % of total lead intake. In average adult consumers, lead dietary exposure ranged from 0.36 to 1.24, up to 2.43 ng kg^−1^ body weight (b.w.) per day in high consumers in Europe (EFSA 2010).

Many authors have reported that human hair mineral analysis is a good marker of environmental pollution. Therefore, using scalp hair as an indicator of the environmental exposure to several trace elements has become a common practice (Özden et al. 2007; Mehra and Juneja 2004; Strumylaite et al. 2004; Shah et al. 2005). The advantage of hair is that it is a storage tissue and retains trace elements over an extended period of time (Laker 1982; Foo et al. 1993). Metal body burden of trace/toxic elements is better reflected in hair than in blood because hair gives a record of relatively long periods, while blood shows momentary levels that fluctuate with time. Furthermore, hair is inert and easier to sample than blood and can be stored without technical problems (Mehra and Juneja 2004). The investigation of Nowak and Chmielnicka (2000) revealed that among the investigated keratinous matrices (hair, nail, tooth), lead content in hair was the mostly meaningful environmental marker of exposure to this metal in the human organism and depended on sex and age.

Human exposure to lead occurs from numerous sources: air, soil, water, and food (D’souza et al. 2011). Lead can enter the human body by inhalation of air, which is polluted with either cigarette smoke, lead-based paint, or with vapors from the combustion of gasoline containing tetraethyl lead or traffic densities. In the work of Serdar et al. (2012), a correlation was observed between the smoking status of family members and levels of toxic trace elements in hair (Zn, Se, B, V, Co, Mo, Mn, Fe, Be, Al, As, Cd, Pb, Hg, Cr, Ag, Be, Ni, Cu, Sn, Sb), where this correlation was more significant with the levels of lead and cadmium. In the work of Özden et al. (2007), it was noticed that school distance from the main street, gender, and number of smokers at home had statistically significant impact on hair lead levels in children (11 and 13 years). Strumylaite et al. (2004) checked the content of lead in human hair from people with various exposure levels in Lithuania. It was found that lead in hair was not related to gender; however, there was a positive relationship between lead in hair and age. Additionally, in men, a positive correlation was found between lead in hair and smoking.

Another potential source of lead is lead-based paints, which were withdrawn from the market for residential use in developed countries because of toxicity concerns. However, paints containing lead are still being used for certain industrial applications (Johnson et al. 2009). Special attention should be also paid to lead content in paints used in children toys. In the work of Weidenhamer (2009), it was found that 12 with 95 of the inexpensive seasonal and holiday products (including primarily Halloween and Easter products) contained lead in excess of the current US regulatory limit of 0.06 % by weight for lead in paints. Another source of lead was in the past in leaded gasoline, which nowadays is banned worldwide. The limitation in the use of lead in gasoline has induced a resulting decrease in atmospheric lead pollution, a remarkable reduction in the lead levels in edible parts of vegetables, as well as a marked decrease in the dietary lead intake of the populations. In the work of Schuhmacher et al. (1996), it was found that a substantial decrease of lead contents in children's hair (53 %) was observed. During the period of 1990–1995, the hair lead contents decreased from 8.8 to 4.1 mg kg^−1^. Wilhelm et al. (2002) examined the lead content in hair of healthy children from three residential areas of Düsseldorf (Germany) with different traffic densities. The lead content in hair for the suburban population was significantly lower than for the population living in the city center. It was also noticed that boys had more lead in hair than girls. In the work of Sanna et al. (2003), it was shown that the content of lead in hair differed between genders and towns at different risks of lead pollution.

The aim of the present study was to investigate the exposure to lead from various sources by determination of mineral composition of human scalp hair. Lead content in human hair was examined by studying the effect of different kinds of food consumed such as cheese, cottage cheese, blue cheese, meat, lettuce, fish, mushrooms, lemon, eggs, yogurt, alcohol, milk, tea, and coffee. Additionally, it was checked whether other factors (e.g., painting the walls, water-supply system, passive smoking, cigarette smoking, amalgam filling, distance from the place of residence to the traffic road, time spent in traffic jams, gas stove) had an impact on lead content in hair.

## Materials and methods

### Sampling and preparation

**Fig. 1:**
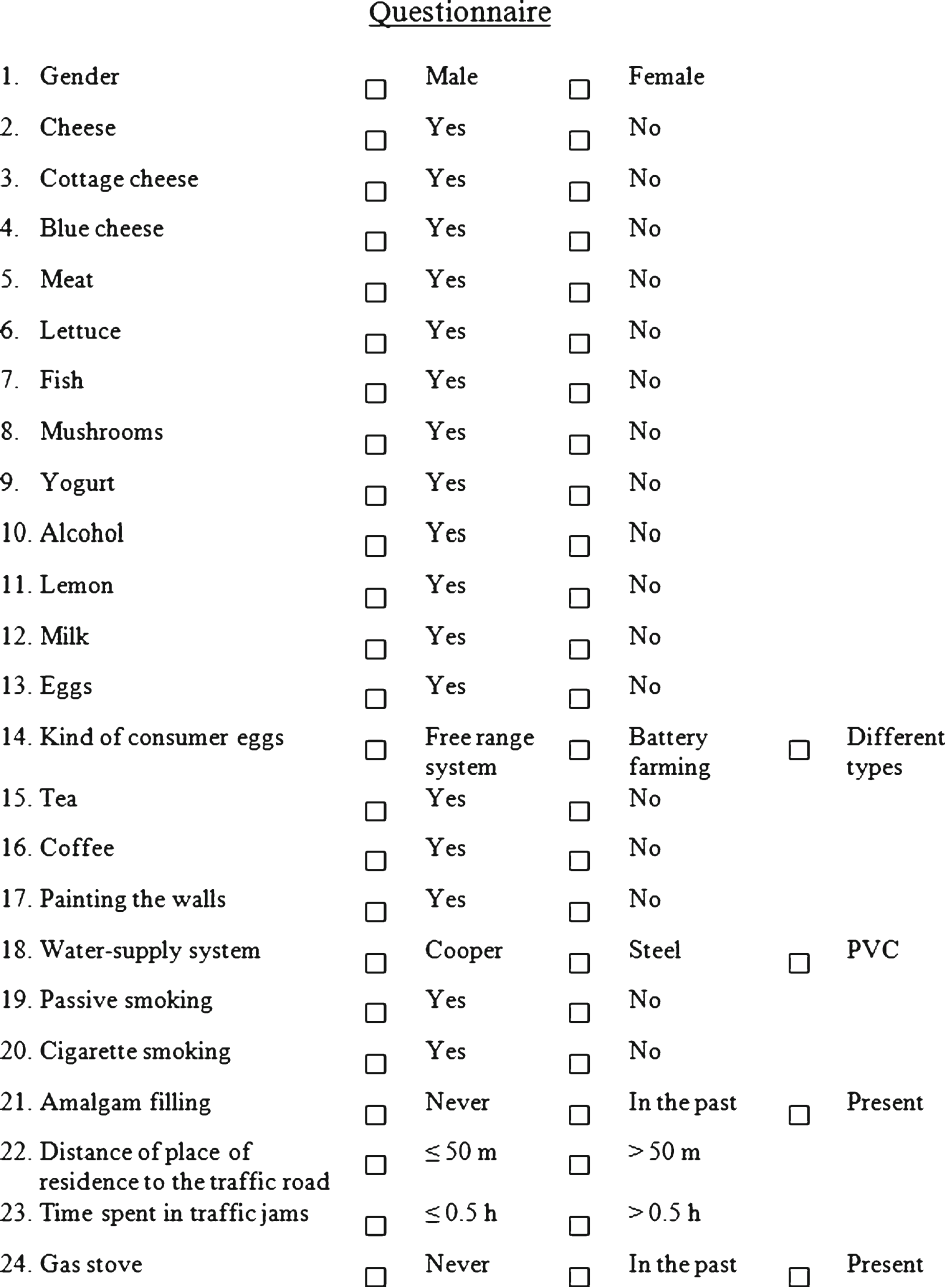
The questionnaire administered to the subjects

Research activities were performed in accordance with the principles laid down in the Helsinki Declaration. The hair samples were provided by volunteers in the years 2009–2010 who filled out a questionnaire, which concerned the individual characteristic (e.g., gender, lifestyle, diet, health condition etc.) (Fig. 1). The present research was carried out on hair sampled from 267 subjects (89 males and 178 females), average age 22 ± 2 years. The population consisted of individuals who lived in Wrocław (a city in Poland, 700,000 citizens) and in the suburbs. The chosen population for mineral hair analysis was uniform when considering environmental and occupational exposure because the experimental group (donor of hair sample) were students of the same department, and the same field of study (Department of Chemistry, Wrocław University of Technology). The participants cut hair (5-cm long) from the nape of the neck directly after four consecutive washings with Johnson's Baby shampoo and drying. For cutting hair, new surgical scissors made from stainless steel were used (Hilbro International (Pvt.) Ltd.). The selection of the shampoo was determined by its composition—among the metal cations, only Na was present. The hair was stored in the paper envelope and was analyzed directly without additional washing steps. The goal was to elaborate easier analytical procedure without steps of washing hair with acids, organic solvents etc., which on one hand are presented in the literature as the method which removes exogenous contamination, but on the other as the source of contamination.

### Analytical methods

A sample (0.5 g) was purified from organic matter with concentrated nitric acid −69 % m/m (5 mL), spectrally pure (Suprapur, Merck) in Teflon bombs in microwave oven Milestone Start D (USA). The selected parameters of the process assured the complete digestion of samples. After mineralization, samples were diluted with re-demineralized water (Millipore Simplicity) to 50 g. Digested samples underwent multielemental analysis. The content of lead was determined by ICP-OES Varian-Vista MPX (Australia), equipped with ultrasonic nebulizer CETAC U5000AT+. The optimized experimental conditions of ICP-OES were as follows: power −1.0 kW, plasma flow −15.0 L min^−1^, nebulizer flow −0.75 L min^−1^, auxiliary flow −2.25 L min^−1^. Lead was determined at the wavelength 220.353 nm. The low limit of detection is 0.0044 mg L^−1^. For the preparation of standard solutions (1.0, 10, 50, 100 mg L^−1^), the multielemental standard (100 mg L^−1^ Astasol®, Prague, Czech Republic) was used. The samples were analyzed in three repeats (the reported results of analyses were arithmetic mean, the relative standard deviation was <5 %). The analyses were carried out in quality management system according to PN-EN ISO/IEC 17025:2005. Quality assurance of the test results was achieved by using certified reference material—Human Hair NCS ZC81002 from the China National Analysis Center. The certified mean content of Pb in NCS reference material–human hair NCS ZC81002 was 7.2 ± 0.7 mg kg^−1^.

### Statistical methods

The results were elaborated statistically by Statistica ver. 9.0. Descriptive statistics (means, standard deviations) were reported. Normality of distribution of experimental results was assessed by Shapiro–Wilk test. On this basis, statistical test was selected, which was used to investigate the significance of differences between the groups. The differences between the groups were investigated with *U* Mann–Whitney test or Kruskal–Wallis test. Results were considered significantly different when *p* < 0.05.

## Results and discussion

In the present paper, the reference value (RV) for lead content in hair for this population was determined (as the values between 10th and 90th percentile). It was assumed that the extreme low and high 10 % of the population reflected deficiency and excess (respectively) of a given element. For the whole population, the range between 10th and 90th percentile was 0.634 mg kg^−1^ −3.94 mg kg^−1^ (reference value should be below 3.94 mg kg^−1^).

In this paper, the mean lead level in hair of the whole examined population (*n* = 267) was 2.01 ± 2.10 mg kg^−1^. This value was much lower than lead content reported in hair in the work of Nowak and Chmielnicka (2000), who examined the exposure to lead during 1990–1997 of inhabitants of Katowice District (Poland's most polluted region) and inhabitants of the Beskid area, which constituted a control group. It was found that lead content in hair of subjects from the first group was 5.7 ± 2.2 mg kg^−1^ (*n* = 184) and in the second group it was 4.8 ± 3.4 mg kg^−1^ (*n* = 440). Similar results were obtained by Nowak (1998), who analyzed the content of lead in hair samples (*n* = 135, age 1–30 years), which were collected between 1990 and 1994 from inhabitants of the Silesian Beskid in the South of Poland (non–industrialized region). The average value was 4.42 ± 2.95 mg kg^−1^. The average value of lead content in hair reported in our study was also much lower than lead content reported in hair in 1997 in the citizens of Gdańsk region (North Poland), which was 5.2 ± 7.9 mg kg^−1^ (*n* = 54, age 47 ± 15) (Hać et al. 1997). Also Trojanowski et al. (2010) analyzed the content of lead in hair collected in 2004–2007 from 106 persons in the age of 16–25 in Central Pomerania in Poland. The average value was 2.84 ± 0.24 mg kg^−1^. The comparison of the results of Polish researchers on the content of lead in hair allows to conclude that human exposure to lead decreased during the last 20 years.

In our paper, the whole population was divided according to gender. It was found that females contained 1.90 ± 1.39 mg kg^−1^ of lead in hair, whereas males: 2.24 ± 3.05 mg kg^−1^. This difference was not statistically significant. Also, Trojanowski et al. (2010) found that hair of Polish females (*n* = 55) contained less lead (2.21 ± 0.24 mg kg^−1^) than males hair (3.51 ± 0.41 mg kg^−1^, *n* = 51). The same tendency was confirmed in the work of Nowak (1998), who found that the content of lead also in Polish female hair (4.32 ± 2.21 mg kg^−1^, *n* = 136) was lower than in male hair (5.70 ± 5.01 mg kg^−1^, *n* = 130). Generally, the tendency to decrease the lead content in hair in both genders was observed.

In Table 1, the exposure to lead from food is presented. Lead may be introduced into food inadvertently during harvesting, processing, packaging, or preparation. The main sources of contamination of food are soil, industrial pollution, agricultural technology, and food processing. Surface contamination of homegrown vegetables, storage cans with lead solder seams, and kitchenware are some of the sources of lead contamination in food. Soil in or adjacent to lead smelters, lead mines, houses painted with lead paint, and urban areas with heavy automobile traffic is likely to contain high concentrations of lead (Tripathi et al. 1989; Clark et al. 2005).

**Table 1:** The effect of different kind of food on lead content in human hair, mg kg^−1^

Kind of food		Mean ± SD
Cheese^*^	Yes (*n* = 242)	2.05 ± 2.14
	No (*n* = 25)	1.63 ± 1.58
Cottage cheese	Yes (190)	2.02 ± 2.22
	No (77)	1.99 ± 1.76
Blue cheese	Yes (119)	1.95 ± 1.58
	No (148)	2.06 ± 2.44
Meat	Yes (98)	1.37 ± 2.39
	No (4)	1.04 ± 0.73
Lettuce	Yes (154)	2.13 ± 2.40
	No (112)	1.87 ± 1.60
Fish	Yes (208)	2.05 ± 2.24
	No (59)	1.90 ± 1.51
Mushrooms	Yes (145)	2.04 ± 2.31
	No (122)	1.98 ± 1.82
Yogurt	Yes (232)	2.02 ± 2.20
	No (35)	1.97 ± 1.24
Alcohol	Yes (231)	2.02 ± 2.15
	No (36)	1.99 ± 1.76
Lemon	Yes (197)	1.84 ± 1.36
	No (70)	2.51 ± 3.37
Milk	Yes (200)	1.85 ± 1.49
	No (67)	2.50 ± 3.28
Eggs	Yes (251)	1.99 ± 2.13
	No (15)	2.35 ± 1.61
Tea	Yes (255)	2.00 ± 2.12
	No (12)	2.33 ± 1.67
Coffee	Yes (196)	1.98 ± 2.25
	No (71)	2.12 ± 1.61

*Statistically significant difference at *p* < 0.05 (determined by nonparametric statistical test: *U* Mann Whitney)

In our work, it was shown that statistically significant difference (*p* = 0.012) was observed between subjects who ate lunches from the university canteen (*n* = 80; 2.18 ± 1.64 mg kg^−1^) and did not (*n* = 187; 1.94 ± 2.26 mg kg^−1^). There are several sources of lead in the consumed food. In the literature, it is indicated that beverages consumed from or stored in lead crystal containers or ceramic vessels glazed with leaded glazes are another potential source of lead intake. The US FDA (Food and Drug Administration) recommend to avoid drinking hot tea, coffee, or other hot acidic beverages from glazed cups and pottery as high temperature can increase dissolution of lead (Sheets 1997). Storage of acidic fruits or beverages in such vessels over a longer period of time could lead to increased lead content. It is also worth mentioning that cooking food in lead-contaminated water has also been shown to result in increased levels of lead in food (Sherlock 1987).

As it was mentioned above, the content of lead in food could be influenced by several factors, e.g., food processing, storage etc. There was no statistically significant differences between subjects, who declared eating canned food in the past (*n* = 44; 2.02 ± 1.30 mg kg^−1^), at present (*n* = 155; 1.98 ± 2.34 mg kg^−1^), and never (*n* = 67; 2.10 ± 1.95 mg kg^−1^). According to Galal‐Gorchev (1991), canned food (lead-soldered cans) could be a potential source of exposure to lead. Typical lead content in canned food could be 200 ng kg^−1^. Another common products such as meat (lead content 50 ng kg^−1^), vegetables (50 ng kg^−1^), eggs (20 ng kg^−1^), or fish (100 ng kg^−1^) are also the potential source of exposure to lead. In our work, statistically significant differences concerned the content of lead in hair of subjects, who declared in the questionnaire eating cheese (subjects who ate cheese had 26 % times higher content of lead in hair, than subjects who did not eat cheese). No statistically significant differences were observed in the case of eating of other kinds of cottage cheese (1.5 % higher lead content in hair of group who ate cheese) and blue cheese (5.3 % lower in group who ate cheese).

The results suggested that eating meat could be a potential source of lead in hair. Subjects, who declared eating meat had 32 % higher content of this toxic element in hair than vegetarians (although this group was very small; *n* = 4). In the work of Trojanowski et al. (2010), the influence of a diet on the content of lead in hair of persons living in Central Pomerania in Poland was examined. In the research, it was found that individuals who consumed both meat and dairy products (*n* = 69) had higher lead content in hair, 3.54 ± 0.45 mg kg^−1^ than individuals who abstained from these products, 2.08 ± 0.17 mg kg^−1^ (*n* = 143). The content of lead in hair of subjects who consumed only meat products was 2.74 ± 0.48 mg kg^−1^ (*n* = 127), whereas of subjects who consumed only dairy products was 3.10 ± 0.30 mg kg^−1^ (*n* = 77). In our work, it was noticed that drinking milk caused reduction of lead content in hair by 26 % in subjects who drank milk. In the literature, it is reported that many toxic effects of lead were associated with the interaction between lead and calcium, which are essential elements. In the work of Kostial et al. (1991), it was assumed that adequate calcium intake might be one of the preventive activity for decreasing lead absorption. In the food monitoring studies performed by Starska et al. (2011), it was found that the average content of lead in milk and liquid processed milk products reported in time period 2006–2007 in Poland was 0.008 mg kg^−1^ (it was much lower than the highest permissible level). The majority of the results (90 %) remained below 0.017 mg kg^−1^. Therefore, milk or other dietary products (e.g., yogurt) are not major sources of exposure to lead in Poland.

According to Galal‐Gorchev (1991), vegetables could be a potential source of lead. In our work, it was found that the subjects who declared eating lettuce had 14 % higher content of lead in hair than subjects who did not eat it. It is known that fresh food may be contaminated by small amounts of lead (as well as other toxic elements) from air deposition, particularly noted for foods such as lettuce, parsley, and mint, which have a very large surface area compared to their mass (Sherlock 1987). Additionally, leafy fresh vegetables grown in lead-containing soils may accumulate lead from dust (ATSDR 2007).

In the case of other common foods and drinks such as fish, mushrooms, and alcohol, the differences between individuals in groups who consumed or not the following products were below 10 %. In the case of subjects who used lemon, a decrease by 27 % in lead content in hair was observed in comparison with the group, who did not use lemon. Dawson et al. (1999) examined 75 adult men (20 to 30 years of age), who smoked one pack of cigarettes per day (minimum). It was found that daily supplementation with 1,000 mg of ascorbic acid resulted in a significant decrease of blood lead levels associated with the general population. It was suggested that ascorbic acid supplementation may provide an economical and convenient method of reducing blood-lead levels, possibly by lowering the intestinal absorption of lead.

Drinking tea and coffee reduced the content of lead in hair by 14 and 7 %, in comparison with the group of subjects who did not drink tea or coffee, but these differences were not statistically significant.

In the questionnaire, we asked the volunteers about the kind of consumed eggs: from free range system, from the battery farming, and from different types. It was found that the content of lead was the highest in the case of consumption of eggs from free range system: 2.43 ± 2.21 mg kg^−1^, then different types, 1.94 ± 2.33 mg kg^−1^ and finally from the battery system, 1.72 ± 1.31 mg kg^−1^. The differences between the examined groups were not statistically significant.

It should be emphasized that for all kinds of consumed food, the content of lead in hair was below the upper value of RV, which was 3.94 mg kg^−1^, what indicates that there is no significant exposure to lead from the diet. Similar observation was also made in the case of other factors, which were listed in Table 2.

**Table 2:** The effect of various factors on the content of lead in hair, mg kg^−1^

Factor		Mean ± SD
Painting the walls	Yes (*n* = 100)	2.21 ± 2.70
	No (*n* = 166)	1.90 ± 1.63
Water-supply system	Cooper (57)	2.55 ± 3.55
	Steel (123)	2.00 ± 1.57
	PVC (84)	1.68 ± 1.27
Passive smoking	Yes (125)	2.02 ± 1.56
	No (81)	1.82 ± 1.32
Cigarette smoking	Yes (60)	2.27 ± 3.49
	No (206)	1.94 ± 1.47
Amalgam filling	Never (153)	1.92 ± 1.62
	In the past (43)	1.97 ± 1.45
	Present (70)	2.25 ± 3.12
Distance to the traffic road	≤50 m (121)	2.01 ± 1.63
	>50 m (146)	2.02 ± 2.42
Time spent in traffic jams	≤ 0.5 h (80)	1.96 ± 1.85
	>0.5 h (131)	1.99 ± 2.33
Gas stove	Never (32)^*^	1.36 ± 1.21
	In the past (33)	1.95 ± 1.41
	Present (201)^*^	2.13 ± 2.28

*Statistically significant difference at *p* < 0.05 (determined by nonparametric statistical test: *U* Mann Whitney and Kruskal–Wallis)

Subjects who had water-supply system made of metal alloys had higher content of lead in hair (52 and 19 % for copper and steel, respectively) than subjects who used water-supply system made of PVC. The differences were not statistically significant. In the literature, it is suggested that sources of lead in tap water could be pipes made of lead, lead-containing solder used to join copper, and other metallic pipes together and plumbing devices made of lead-containing brass (e.g., water meters, valves, components in water fountains, and in faucets) (Triantafyllidou and Edwards 2012). Solder containing 40–50 % lead by weight was used in US buildings before 1986. After this time, only lead-free solder, containing less than 0.2 % lead by weight, was allowed in buildings. Brass and bronze are copper alloys which contain lead. Typically, lead content in the brass is from 1.5 to 8 % by weight (Triantafyllidou and Edwards 2012). High levels of lead can be present as impurities in the zinc coating used to protect steel pipes (Schock et al. 1996).

Beside food, toxic metals can also enter the human body by inhalation. In Table 2, the effect of various factors on the content of lead in hair is presented. Other source of exposure to lead could be cigarettes which contain 0.6–17 μg lead (Özden et al. 2007). According to Galażyn-Sidorczuk (2008), the mean lead content in the cigarettes produced in Poland was 0.6853 ± 0.0746 μg per cigarette with an average 11 % of lead released to cigarette smoke. In our work, the content of lead was 17 % higher in hair of subjects who declared smoking versus non-smoking but the difference was not statistically significant. The increased lead level in hair of smoking subjects in this study was lower than the results reported by Mortada et al. (2002), for 28–40 years male (51 %; 8.09 ± 0.86 and 5.37 ± 3.41 mg kg^−1^ for smokers and nonsmokers, respectively), Massadeh et al. (2011), for 15–30 years male (51 %; 7.3 and 11 mg kg^−1^) and for 31–60 years male (58 %; 5.9 and 9.3 mg kg^−1^), Sukumar and Subramanian (2003), for 7–30 years students (87 %; 7.5 ± 1.3 and 4.0 ± 0.3 mg kg^−1^), Cai (2011), for 20–24 students (approximately three times; 13.92 and 5.14 mg kg^−1^), Kazi et al. (2009) for 25–55 subjects (27 %; 8.7 ± 1.8 and 6.85 ± 1.55 mg kg^−1^). Additionally, smoking subjects who consumed lemons had over two times lower lead level as compared to smoking subjects who did not eat lemons (*n* = 41; 1.56 ± 1.12 mg kg^−1^ and *n* = 19; 3.79 ± 5.80 mg kg^−1^, respectively). It may be associated with vitamin C and E which have been used as chelating agents for the treatment of chronic lead poisoning (Flora et al. 2013). Increased level of lead in hair of subjects who were passive smokers (11 % higher content of lead in hair than subjects who were not exposed to cigarette smoke) was also observed. The differences between the examined groups were not statistically significant. In the work of Serdar et al. (2012), it was showed that the children living in smoking households had more than two times higher content of lead in hair than the children living in non-smoking households. There was a significant correlation between the smoking level of mothers and the level of toxic elements including lead in hair.

Distance from the traffic road and time spent in traffic jams had no influence on the content of lead in hair of the studied population. The lack of differences could result from the fact that in Poland the petroleum with tetraethyl lead was withdrawn from the market in 2005 (Hofman and Wachowski 2010). Schuhmacher et al. (1996) showed that hair lead levels decreased from 8.8 to 4.1 mg/kg during the period of 1990–1995 together with reduced consumption of leaded gasoline.

Additionally, it was found that subjects who declared painting the walls had 16 % higher content of lead in hair than subjects who did not declare painting. It may be associated with scraping and burning of old lead-based paints off the walls and window frames during renovation of old houses (Howarth 2012).

Amalgam filling could be a potential source of lead in hair. Subjects with amalgam filling at present had 17 % higher content of lead in hair than subjects who never had amalgam filling. Subjects who declared using gas stove at present or in the past had higher level of lead in hair (57 and 43 %, respectively) than subjects who never used gas stove. We have found no information in the literature if natural gas could be the source of lead.

## Conclusion

The present paper investigated the exposure to lead from various sources based on the questionnaire survey results. It was found that the content of lead in hair depended on dietary factors and other factors such as painting the walls, water-supply system, passive smoking, cigarette smoking, amalgam filling, distance from the traffic road, time spent in traffic jams, and gas stove. However, the content of lead in hair was below reference value. It was found that statistically significant difference was observed between the subjects who ate from the university canteen and those who did not and the ones' who declared in the questionnaire eating cheese. It is worth mentioning that in our work it was observed that drinking milk and using lemon caused reduction of lead content in hair. Another interesting observation was that subjects who had water-supply system made of metal alloys had higher content of lead in hair than subjects who used water-supply system made of PVC. Hair mineral analysis combined with a questionnaire survey was found useful in determination of human exposure to lead from different sources.

## References

[CR1] ATSDR (Agency for Toxic Substances and Disease Registry) (2007). Toxicological profile for lead. Atlanta. Available from: http://www.atsdr.cdc.gov/toxprofiles/tp13.pdf (Access 2nd February 2013).

[CR2] Cai Y (2011). Determination of select trace elements in hair of college students in Jinzhou, China. Biological Trace Element Research.

[CR3] Clark CS, Thuppil V, Clark R, Sinha S, Menezes G, D’Souza H (2005). Lead in paint and soil in Karnataka and Gujarat (India). Journal of Occupational Environmental Hygiene.

[CR4] D’souza HS, Dsouza SA, Menezes G, Venkatesh T (2011). Diagnosis, evaluation, and treatment of lead poisoning in general population. Indian Journal of Clinical Biochemistry.

[CR5] Dawson EB, Evans DR, Harris WA, Teter MC, McGanity WJ (1999). The effect of ascorbic acid supplementation on the blood lead levels of smokers. Journal of American College of Nutrition.

[CR6] EFSA (European Food Safety Authority). (2010). Panel on Contaminants in the Food Chain (CONTAM). Scientific Opinion on Lead in Food. EFSA Journal, 8, 1570.

[CR7] Flora G, Gupta D, Tiwari A (2013). Preventive efficacy of bulk and nanocurcumin against lead-induced oxidative stress in mice. Biological Trace Element Research.

[CR8] Foo SC, Khoo NY, Heng A, Chua LH, China SE, Ong CN (1993). Metals in hair as biological indices for exposure. International Archives of Occupational and Environmental Health.

[CR9] Galal‐Gorchev H (1991). Dietary intake of pesticide residues: cadmium, mercury, and lead. Food Additives and Contamination.

[CR10] Galażyn-Sidorczuk M, Brzóska MM, Moniuszko-Jakoniuk J (2008). Estimation of Polish cigarettes contamination with cadmium and lead, and exposure to these metals via smoking. Environmental Monitoring and Assessment.

[CR11] Hać E, Czarnowski W, Gos T, Krechniak J (1997). Lead and fluoride content in human bone and hair in the Gdańsk region. Science of the Total Environment.

[CR12] Hofman M, Wachowski L (2010). Soil concentrations of platinum and lead along the main exit routes from the city of Poznań. Ochrona Środowiska.

[CR13] Howarth D (2012). Lead exposure. Implications for general practice. Australian Family Physician.

[CR14] Johnson S, Saikia N, Sahu R (2009). Lead in Paints.

[CR15] Kazi TG, Jalbani N, Kazi N, Arain MB, Jamali MK, Afridi HI (2009). Estimation of toxic metals in scalp hair samples of chronic kidney patients. Biological Trace Element Research.

[CR16] Kostial K, Dekanić D, Telišman S, Blanusa M, Duvancić S, Prpić-Majić D (1991). Dietary calcium and blood lead levels in women. Biological Trace Element Research.

[CR17] Laker M (1982). On determining trace element levels in man, the uses of blood and hair. Lancet.

[CR18] Li Y, Zhang B, Li H, Yang L, Ye B, Wang W, Rosenberg M (2013). Biomarkers of lead exposure among a population under environmental stress. Biological Trace Element Research.

[CR19] Massadeh A, El-Rjoob AW, Samadi H (2011). Lead, cadmium, copper, zinc, iron, and calcium in human hair as a function of gender, age, smoking, and hair dyeing. Toxicological and Environmental Chemistry.

[CR20] Mehra R, Juneja M (2004). Biological monitoring of lead and cadmium in human hair and nail and their correlations with biopsy materials, age, and exposure. Indian Journal of Biochemistry and Biophysics.

[CR21] Mielkel HW, Reagan PL (1998). Soil is an important pathway of human lead exposure. Environmental Health Perspectives.

[CR22] Mortada WI, Sobh MA, El-Defrawy MM, Farahat SE (2002). Reference intervals of cadmium, lead, and mercury in blood, urine, hair, and nails among residents in Mansoura City, Nile Delta, Egypt. Environmental Research Section A.

[CR23] Nowak B (1998). Contents and relationship of elements in human hair for a non-industrialized population in Poland. Science of the Total Environment.

[CR24] Nowak B, Chmielnicka J (2000). Relationship of lead and cadmium to essential elements in hair, teeth, and nails of environmentally exposed people. Ecotoxicology and Environmental Safety.

[CR25] Özden TA, Gökçay G, Ertem HV, Suoglu OD, Kilic A, Sokucu S (2007). Elevated hair levels of cadmium and lead in school children exposed to smoking and in highways near schools. Clinical Biochemistry.

[CR26] Pizzol M, Thomsen M, Andersen MS (2010). Long-term human exposure to lead from different media and intake pathways. Science of the Total Environmental.

[CR27] Sanna E, Liguori A, Palmas L, Soro MR, Floris G (2003). Blood and hair lead levels in boys and girls living in two Sardinian towns at different risks of lead pollution. Ecotoxicology and Environmental Safety.

[CR28] Schock MR, Wagner I, Oliphant RJ (1996). Corrosion and solubility of lead in drinking water in internal corrosion of water distribution systems.

[CR29] Schuhmacher M, Bellés M, Rico A, Domingo JL, Corbella J (1996). Impact of reduction of lead in gasoline on the blood and hair lead levels in the population of Tarragona Province, Spain, 1990–1995. Science of the Total Environment.

[CR30] Serdar MA, Akin BS, Razi C, Akin O, Tokqoz S, Kenar L (2012). The correlation between smoking status of family members and concentrations of toxic trace elements in the hair of children. Biological Trace Element Research.

[CR31] Shah MH, Shaheen N, Jaffar M, Khalique A, Tariq SR, Manzoor S, Ahmed W (2005). Age and gender-based comparison of nickel content of scalp hair of edible oil- and hydrogenated oil-consuming populations. Human and Ecological Risk Assessment.

[CR32] Sheets RW (1997). Extraction of lead, cadmium, and zinc from overglaze decorations on ceramic dinnerware by acidic and basic food substances. Science of the Total Environment.

[CR33] Sherlock JC (1987). Lead in food and the diet. Environmental Geochemistry and Health.

[CR34] Starska K, Wojciechowska-Mazurek M, Mania M, Brulińska-Ostrowska E, Biernat U, Karłowski K (2011). Noxious elements in milk and milk products in Poland. Polish Journal of Environmental Studies.

[CR35] Strumylaite L, Ryselis S, Kregzdyte R (2004). Content of lead in human hair from people with various exposure levels in Lithuania. International Journal of Hygiene and Environmental Health.

[CR36] Sukumar A, Subramanian R (2003). Elements in the hair of non-mining workers of a lignite open mine in Neyveli. Industiral Health.

[CR37] Triantafyllidou S, Edwards M (2012). Lead (Pb) in tap water and in blood: implications for lead exposure in the United States. Critical Reviews in Environmental Science and Technology.

[CR38] Tripathi RM, Khandekar RN, Raghunath R, Mishra UC (1989). Assessment of atmospheric pollution from toxic heavy metals in two cities in India. Atmospheric Environment.

[CR39] Trojanowski P, Trojanowski J, Antonowicz J, Bokiniec M, Trojanowski J (2010). Lead and cadmium content in human hair in central Pomerania (northern Poland). Journal of Elementology.

[CR40] Weidenhamer JD (2009). Lead contamination of inexpensive seasonal and holiday products. Science of the Total Environment.

[CR41] WHO (World Health Organization) (1995). Environmental Health Criteria 165.

[CR42] Wilhelm M, Pesch A, Rostek U, Begerow J, Schmitz N, Idel H (2002). Concentrations of lead in blood, hair, and saliva of German children living in three different areas of traffic density. Science of the Total Environment.

[CR43] Ziemacki G, Viviano G, Merli F (1989). Heavy metals: sources and environmental presence. Annali dell’Istituto Superiore di Sanita.

